# Temporal trends in the presentation of cardiovascular and cerebrovascular emergencies during the COVID-19 pandemic in Germany: an analysis of health insurance claims

**DOI:** 10.1007/s00392-020-01723-9

**Published:** 2020-08-04

**Authors:** Moritz Seiffert, Fabian J. Brunner, Marko Remmel, Götz Thomalla, Ursula Marschall, Helmut L’Hoest, Laura Acar, Eike S. Debus, Stefan Blankenberg, Christian Gerloff, Christian-Alexander Behrendt

**Affiliations:** 1 Department of Cardiology, University Heart and Vascular Center UKE Hamburg, University Medical Center Hamburg-Eppendorf, Hamburg, Germany; 2 Department of Neurology, University Medical Center Hamburg-Eppendorf, Hamburg, Germany; 3 BARMER, Wuppertal, Germany; 4 Research Group GermanVasc, Department of Vascular Medicine, University Heart and Vascular Center UKE Hamburg, University Medical Center Hamburg-Eppendorf, Hamburg, Germany; 5 German Center for Cardiovascular Research (DZHK), Partner Site Hamburg/Kiel/Lübeck, Hamburg, Germany

**Keywords:** COVID-19, Pandemic, Health services research, Myocardial infarction, Stroke, Emergencies

## Abstract

**Aims:**

The first reports of declining hospital admissions for major cardiovascular emergencies during the COVID-19 pandemic attracted public attention. However, systematic evidence on this subject is sparse. We aimed to investigate the rate of emergent hospital admissions, subsequent invasive treatments and comorbidities during the COVID-19 pandemic in Germany.

**Methods and results:**

This was a retrospective analysis of health insurance claims data from the second largest insurance fund in Germany, BARMER. Patients hospitalized for acute myocardial infarction, acute limb ischemia, aortic rupture, stroke or transient ischemic attack (TIA) between January 1, 2019, and May 31, 2020, were included. Admission rates per 100,000 insured, invasive treatments and comorbidities were compared from January–May 2019 (pre-COVID) to January–May 2020 (COVID). A total of 115,720 hospitalizations were included in the current analysis (51.3% females, mean age 72.9 years). Monthly admission rates declined from 78.6/100,000 insured (pre-COVID) to 70.6/100,000 (COVID). The lowest admission rate was observed in April 2020 (61.6/100,000). Administration rates for ST-segment elevation myocardial infarction (7.3–6.6), non-ST-segment elevation myocardial infarction (16.8–14.6), acute limb ischemia (5.1–4.6), stroke (35.0–32.5) and TIA (13.7–11.9) decreased from pre-COVID to COVID. Baseline comorbidities and the percentage of these patients treated with interventional or open-surgical procedures remained similar over time across all entities. In-hospital mortality in hospitalizations for stroke increased from pre-COVID to COVID (8.5–9.8%).

**Conclusions:**

Admission rates for cardiovascular and cerebrovascular emergencies declined during the pandemic in Germany, while patients’ comorbidities and treatment allocations remained unchanged. Further investigation is warranted to identify underlying reasons and potential implications on patients’ outcomes.

**Graphic abstract:**

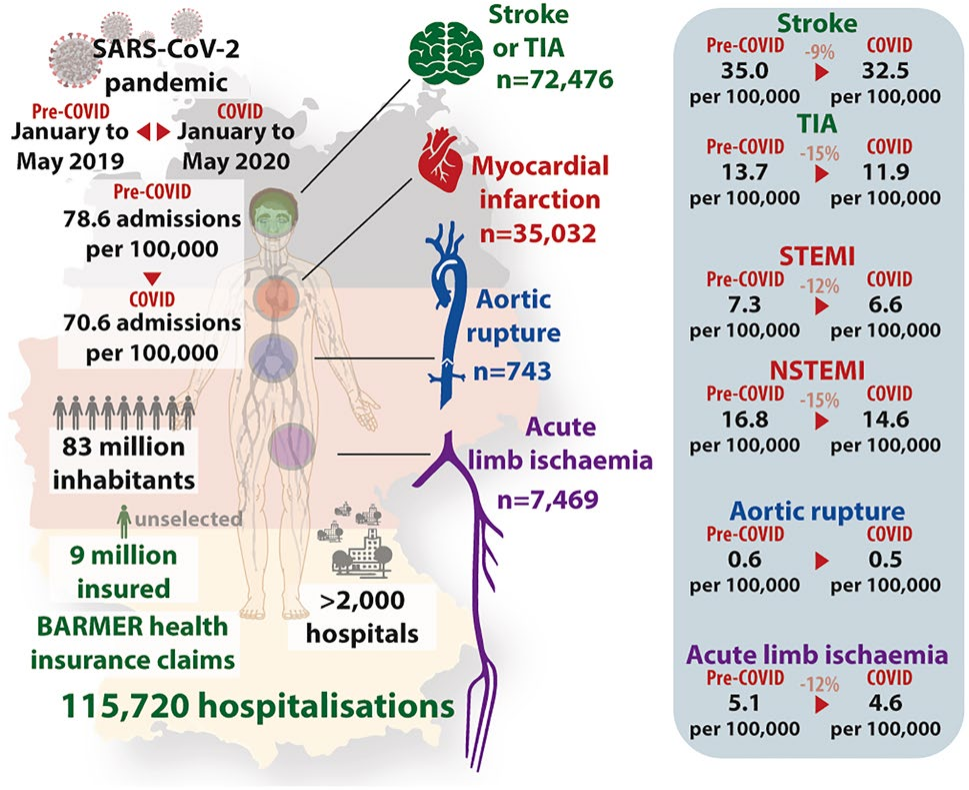

**Electronic supplementary material:**

The online version of this article (10.1007/s00392-020-01723-9) contains supplementary material, which is available to authorized users.

## Introduction

Since its outbreak in Wuhan, Hubei Province, China, in December 2019, the novel SARS coronavirus (SARS-CoV-2) has spread rapidly, causing an outbreak of acute and severe respiratory illness worldwide [1]. The first disease outbreak news from the World Health Organization (WHO) had been issued January 5, 2020. After a rapid spread of SARS-CoV-2 in Italy [2] and other European regions, several countries issued strict infection control measures. Elective in-hospital procedures were widely cancelled or postponed to provide additional capacities for the treatment of COVID-patients in Germany, starting in March 2020 [3, 4].

While an association between acute viral respiratory disease and subsequent cardiovascular events had been described for other diseases (e.g., influenza) [5, 6], several centers reported a decline in hospital admissions for acute cardiovascular and cerebrovascular emergencies during the COVID pandemic [7–19]. Since acute cardiovascular and cerebrovascular diseases remain leading causes for morbidity and mortality, further investigation of this concerning trend is warranted to identify potential implications for both health-care professionals and regulators.

This analysis sought to determine trends in admission rates, invasive treatments, and comorbidities of inpatients treated for cardiovascular and cerebrovascular emergencies during the SARS-CoV-2 pandemic in Germany.

## Methods

### Study design

This was a retrospective analysis of routinely collected health insurance claims data. Based on their primary or admission diagnosis, we included all patients with inpatient treatment between January 1, 2019, and May 31, 2020, for cardiovascular and cerebrovascular emergencies. These included (1A) ST-segment elevation myocardial infarction (STEMI), (1B) non-ST-segment elevation myocardial infarction (NSTEMI), (2) acute limb ischemia, (3) acute aortic rupture, (4A) acute stroke and (4B) transient ischemic attack (TIA) (for detailed coding see Supplemental Table 1).

Data collected during the pandemic (COVID: January through May 2020) were compared to a control period in the previous year (pre-COVID: January through May 2019). The primary study end point was the absolute number of monthly hospitalizations and the corresponding admission rate per 100,000 insured inhabitants. Secondary end points included the absolute number and proportion of invasive procedures provided to these patients (for detailed coding see Supplemental Table 1), as well as all-cause mortality during the hospital stay and the proportion of relevant comorbidities and socio-demographic variables.

### Sample and database

The longitudinal data of Germany’s second-largest insurance fund, BARMER, includes the outpatient and inpatient medical care provided to up to 9.4 million (from 2008 to 2020) German citizens (13.2% of Germany’s population) involving more than 24 million hospitalizations between January 1, 2008, and May 31, 2020. The BARMER cohort is similar to Western European countries and has been widely used for research projects before [20, 21]. A regular random sample validation of internal and external validity is performed by the Medical Service of the Health Funds (MDK) in Germany, and various peer-reviewed validation studies have been published before [22, 23].

### Study variables

The diagnoses and comorbidities routinely collected in health insurance claims data follow the commonly accepted international standard for reporting diseases and health conditions using World Health Organization (WHO) International Classification of Diseases in its 10th revision of the German Modification (ICD-10-GM) and Operations and Procedures Codes (OPS) as a German adaptation of the International Classification of Procedures in Medicine (ICPM) by WHO.

The primary diagnosis of the hospital case was used to discriminate between the cardiovascular or cerebrovascular emergencies. In approximately 0.6% of the most current hospitalizations (in May 2020), the admission diagnosis was utilized on a supplementary basis if no primary diagnosis was available. In addition to age (in years) and gender (dichotomized), we used the following ICD-10 codes to identify diabetes (E10*, E11*, E12*, E13*, E14*), hypertension (I10*, I11*, I12*, I13*, I14*, I15*), heart failure (I50*), atrial fibrillation (I48*), chronic ischemic heart disease (I25*), obesity (E66*), chronic renal disease (N18*), chronic obstructive pulmonary disease (COPD, J44*), and cancer (C00-97*) as comorbidities among the study sample. All-cause mortality was provided during the index hospital stay.

### Statistical analysis

We summarized the baseline characteristics of the patients with means for age and with percentages and 95% confidence interval (CI) for discrete variables. A comparison of 95% CI and Chi square test was used to test for differences. For Table 1, we compared symmetric samples from January 01, 2019, to May 31, 2019 (pre-COVID), vs. January 01, 2020, to May 31, 2020 (COVID), to adjust for seasonal effects. Missing data (0.6%) were handled by exclusion.

**Table 1:** Admission rates and baseline characteristics and comorbidities of pre-COVID vs. COVID groups (January to May 2019 vs. January to May 2020)

	STEMI		NSTEMI		Acute limb ischemia		Aortic rupture		Stroke		TIA	
	Pre-COVID	COVID	Pre-COVID	COVID	Pre-COVID	COVID	Pre-COVID	COVID	Pre-COVID	COVID	Pre-COVID	COVID
No. of patients, n	3,350	2,940	7,682	6,518	2,331	2,041	257	215	15,969	14,546	6,252	5,342
Admission rate per 100,000	7.3	6.6	16.8	14.6	5.1	4.6	0.6	0.5	35.0	32.5	13.7	11.9
Absolute change (%)	− 12.2^#^		− 15.2^#^		− 12.4^#^		− 16.3 (n.s.)		− 8.9^#^		− 14.6^#^	
February (%)	− 0.5		− 9.0		− 9.5		− 3.8		+ 0.1		− 7.5	
March (%)	− 20.8		− 19.6		− 0.2		− 7.3		− 5.4		− 22.6	
April (%)	− 16.3		− 21.5		− 22.5		− 38.2		− 17.8		− 30.2	
Mai (%)	− 9.0		− 18.6		− 26.0		− 46.9		− 21.2		− 12.1	
Fraction^a^ of interventional/ open-surgical treatment, %	84.7	86.3	58.0	60.5	81.9	82.8	51.5	56.7	18.4	19.1	2.1	2.2
In-hospital mortality, %	11.9 (10.8–13.0)	12.1 (11.0–13.4)	6.0 (5.5–6.6)	5.6 (5.1–6.2)	5.6 (4.7–6.6)	5.4 (4.5–6.5)	44.4 (38.2–50.7)	37.2 (30.7–44.0)	8.5^#^ (8.1–9.0)	9.8^#^ (9.3–10.3)	0.3 (0.2–0.5)	0.5 (0.3–0.7)
Female, %	38.2	38.7	40.4	41.8	51.4	53.1	40.9	38.6	55.3	55.5	59.3	59.4
Mean age, years	68	68	74	73	73	73	76	74	75	76	74	74
Diabetes	22.2 (20.8–23.7)	23.3 (21.8–24.9)	31.3 (30.3–32.4)	31.4 (30.3–32.6)	26.2 (24.4–28.1)	27.2 (25.3–29.2)	10.1 (6.7–14.5)	10.7 (6.9–15.6)	25.4 (24.7–26.1)	25.5 (24.8–26.2)	20.3 (19.3–21.3)	20.7 (19.6–21.8)
Obesity	6.5 (5.6–7.3)	7.5 (6.6–8.5)	7.5 (6.9–8.1)	7.4 (6.7–8.0)	5.7 (4.8–6.7)	5.6 (4.6–6.7)	4.3 (2.2–7.5)	3.7 (1.6–7.2)	4.7 (4.4–5.0)	4.0 (3.7–4.3)	3.5 (3.1–4.0)	3.3 (2.8–3.8)
Hypertension	67.6 (65.9–69.1)	68.7 (67.0–70.4)	77.3^#^ (76.4–78.3)	79.3^#^ (78.3–80.3)	71.6^#^ (69.7–73.4)	75.6^#^ (73.6–77.4)	59.9 (53.7–66.0)	60.0 (53.1–66.6)	76.6^#^ (75.9–77.2)	77.8^#^ (77.2–78.5)	72.8 (71.7–73.9)	73.5 (72.3–74.7)
Ischemic heart disease	93.2 (92.3–94.1)	94.1 (93.2–94.9)	87.8 (87.1–88.6)	88.1 (87.3–88.9)	26.4 (24.7–28.3)	27.8 (25.9–29.8)	23.7 (18.7–29.4)	19.1 (14.1–25.0)	14.7 (14.2–15.3)	14.6 (14.0–15.2)	13.4 (12.6–14.3)	13.0 (12.1–13.9)
Atrial fibrillation	17.9 (16.6–19.3)	15.3 (14.0–16.7)	26.4 (25.4–27.4)	26.4 (25.3–27.5)	23.1 (21.4–24.9)	24.6 (22.7–26.5)	29.6 (24.1–35.6)	29.8 (23.7–36.4)	30.6 (29.9–31.4)	30.3 (29.5–31.0)	22.4 (21.4–23.5)	21.6 (20.5–22.7)
Heart failure	39.9 (38.2–41.5)	39.6 (37.8–41.4)	40.5 (39.4–41.6)	40.1 (38.8–41.2)	13.4 (12.1–14.9)	13.6 (12.2–15.2)	17.9 (13.4–23.1)	18.1 (13.2–24.0)	10.9 (10.4–11.4)	11.2 (10.7–11.7)	6.7 (6.1–7.4)	6.8 (6.2–7.5)
COPD	5.2 (4.4–6.0)	4.9 (4.2–5.8)	8.8 (8.2–9.4)	7.8 (7.2–8.5)	10.3 (9.1–11.6)	12.0 (10.6–13.4)	10.1 (6.7–14.5)	7.0 (4.0–11.3)	4.7 (4.4–5.0)	5.0 (4.6–5.3)	4.1 (3.7–4.7)	3.3 (2.9–3.9)
Chronic renal disease	13.5 (12.3–14.7)	12.1 (11.0–13.3)	24.3 (23.4–25.3)	23.5 (22.5–24.5)	23.8 (22.1–25.6)	24.9 (23.0–26.8)	20.6 (15.9–26.1)	16.3 (11.6–21.9)	15.3 (14.7–15.8)	14.7 (14.2–15.3)	11.1 (10.4–11.9)	12.4 (11.6–13.3)
Cancer	1.7 (1.3–2.2)	1.7 (1.3–2.3)	2.6 (2.3–3.0)	2.2 (1.9–2.6)	3.4 (2.7–4.2)	2.8 (2.2–3.7)	3.1 (1.4–6.0)	5.6 (2.9–9.6)	3.4 (3.2–3.7)	3.3 (3.0–3.6)	1.9 (1.6–2.2)	1.8 (1.4–2.2)

Proportions for comorbidities are presented as % with 95% confidence interval in parentheses

^#^Statistically significant differences (*p* < 0.05). *n.s.* not significant

^a^Fraction denotes the percentage of patients admitted for cardiovascular and cerebrovascular emergencies, who underwent respective interventional or open-surgical treatment. STEMI: ST-segment elevation myocardial infarction; NSTEMI: non-STEMI; TIA: transient ischemic attack; COPD: chronic obstructive pulmonary disease

As additional sensitivity analysis, we compared the study variables using shorter time periods (e.g., March 2019 vs. March 2020, April 2019 vs. April 2020, May 2019 vs. May 2020). Besides, we determined the admission rates using either both primary and admission diagnosis vs. only primary diagnosis (only reimbursed cases).

Data processing was performed with software SAS version 9.04 (SAS Institute, North Carolina, USA) and SPSS version 25 (IBM Corporation, New York, USA), visualization was performed with software Adobe Illustrator version 24.1.2 (Adobe, California, USA).

## Results

We identified 115,720 hospitalizations (mean age 72.9 years, 51.3% females, 95% CI 51.0% to 51.6%) for cardiovascular or cerebrovascular emergencies between January 1, 2019, and May 31, 2020 (monthly mean: 6,807 patients) (Table 1). Among all hospitalizations, a total of 8,202 deaths occurred. The monthly hospital admission rate for cardiovascular or cerebrovascular emergencies declined from a maximum of 83.8 per 100,000 in January 2019 to a minimum of 61.6 per 100,000 in April 2020, which increased to 67.1 per 100,000 in May 2020 (mean: 75.2 per 100,000).

**Fig. 1:**
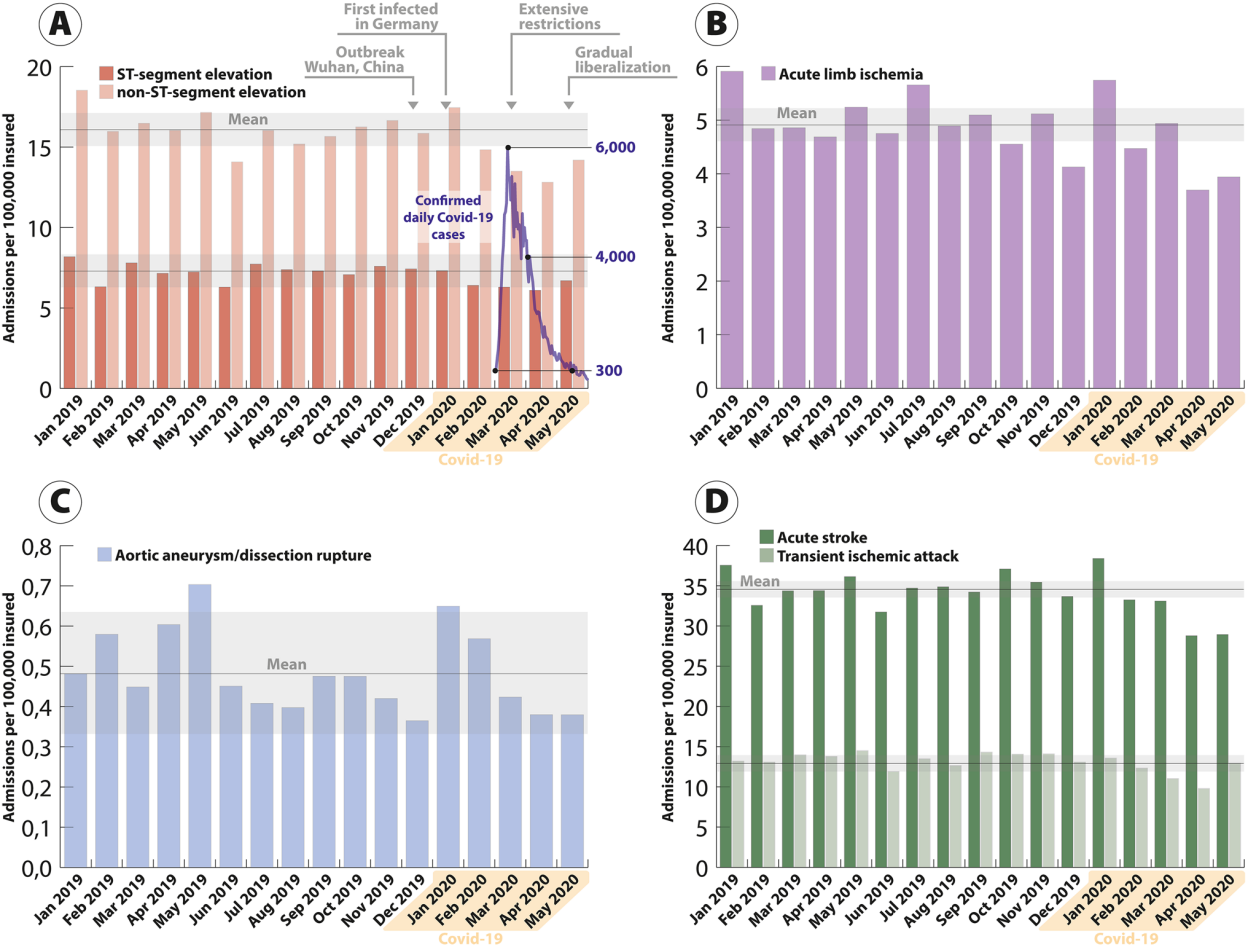
Monthly admission rates for cardiovascular and cerebrovascular emergencies per 100,000 insured in Germany for ST-segment elevation myocardial infarction (dark red) and non-ST-segment elevation myocardial infarction (light red) (**a**) for acute limb ischemia (**b**), aortic rupture (**c**), and acute stroke (dark green) and transient ischemic attack (light green) (**d**) between January 2019 and May 2020. The gray line with shade denotes the annual mean for 2019 with a corresponding limit of agreement

Comparing pre-COVID to COVID time frames, overall monthly admission rates declined from 78.6 per 100,000 to 70.6 per 100,000. This was observed across all strata (Fig. 1): admission rates per 100,000 during COVID compared to pre-COVID decreased for STEMI (7.3 vs. 6.6, − 12.2% percentage points, p.p.), NSTEMI (16.8 vs. 14.6, − 15.2% p.p.), acute limb ischemia (5.1 vs. 4.6, − 12.4% p.p.), stroke (35.0 vs. 32.5, − 8.9% p.p.), and TIA (13.7 vs. 11.9, − 14.6% p.p.). No relevant differences were observed for aortic ruptures (0.6 vs. 0.5) (Fig. 1 and Table 1). The lowest admission rates were noted in April 2020 (61.6 per 100,000) with declines in STEMI (− 16.3%), NSTEMI (− 21.5%), acute limb ischemia (− 22.5%), stroke (− 17.8%), and TIA (− 30.2%) compared to the respective rates in April 2019. For May 2020, slowly recovering admission rates were observed for NSTEMI, STEMI, ALI, stroke, and TIA compared to April 2020 (Fig. 1). The admission rates of NSTEMI and STEMI and the corresponding numbers of daily SARS-CoV-2 infections in Germany from January through May 2020 are depicted in Fig. 1a.

Comorbidities, cardiovascular risk profiles and socio-demographic variables were similar among patients admitted during COVID compared to pre-COVID eras (Table 1).

Besides patients admitted for stroke (8.5–9.8%), the in-hospital mortality was similar among patients admitted during COVID compared to pre-COVID eras (Table 1).

**Fig. 2:**
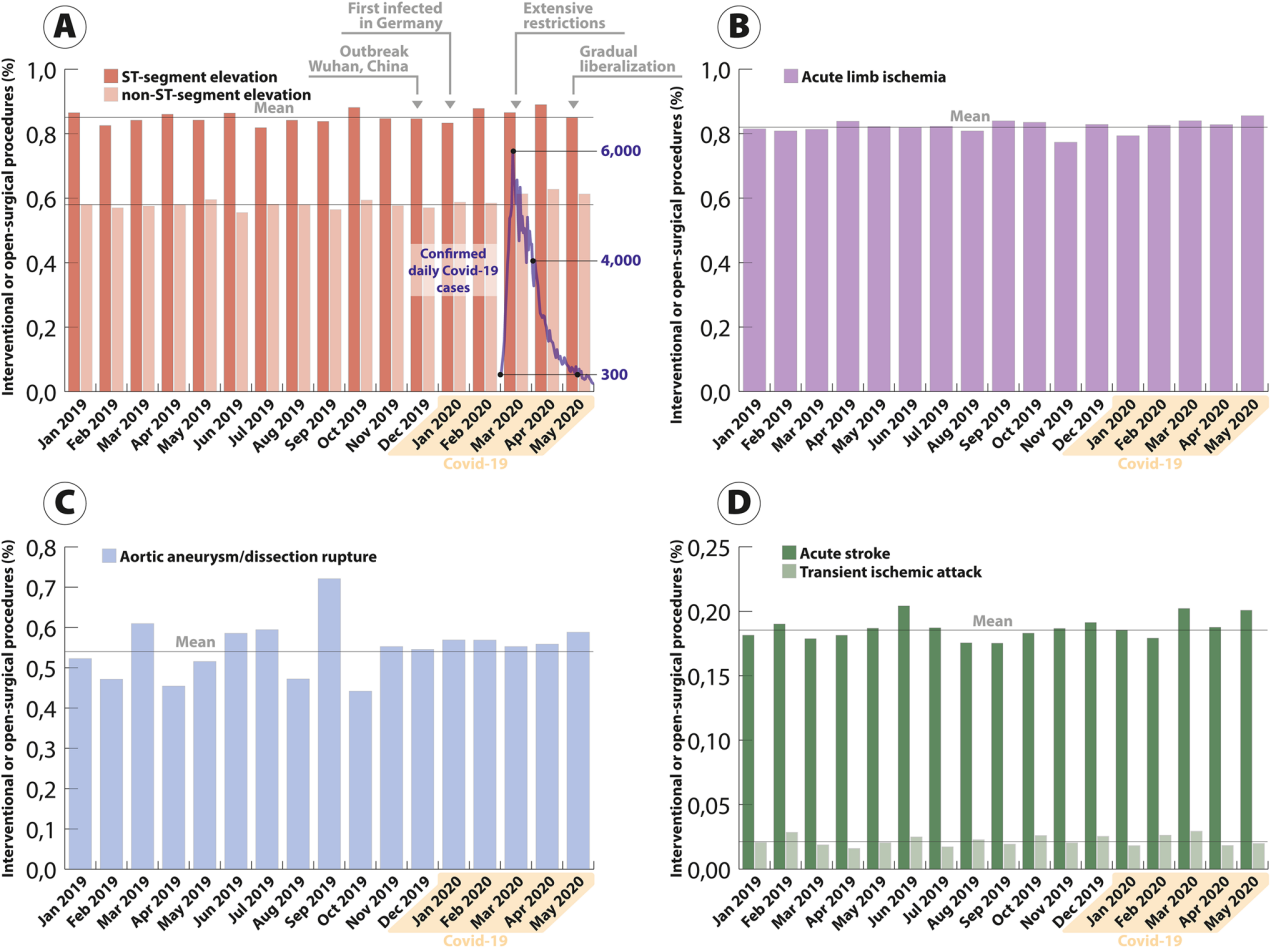
Percentage of patients admitted for cardiovascular and cerebrovascular emergencies, who underwent respective interventional or open-surgical treatment. Rates per month are given for ST-segment elevation myocardial infarction (dark red) and non-ST-segment elevation myocardial infarction (light red) (**a**) for acute limb ischemia (**b**), aortic rupture (**c**), and acute stroke (dark green) and transient ischemic attack (light green) (**d**) between January 2019 and May 2020. The gray line denotes the annual mean for 2019

The percentage of patients admitted for cardiovascular or cerebrovascular emergencies, who underwent interventional or open-surgical procedures during the hospital stay, were similar between pre-COVID and COVID periods for STEMI (84.7–86.3%), NSTEMI (58.0–60.5%), acute limb ischemia (81.9–82.8%), aortic rupture (51.5–56.7%), stroke (18.4–19.1%), and TIA (2.1–2.2%) (Fig. 2 and Table 1). All sensitivity analyses were confirmative (not shown).

## Discussion

This analysis of a large dataset of routinely collected health insurance claims demonstrated a marked decrease in hospital admission rates for several cardiovascular and cerebrovascular emergencies during the COVID-19 pandemic in Germany. These patients’ comorbidities and the percentage of them, who were treated invasively, remained unchanged.

A concerning decline of more than 40% in hospital admissions for acute coronary syndromes after the COVID-19 outbreak had been reported in smaller series of severely affected regions [9, 11–14, 18, 24]. We observed reductions of 12.2% in patients presenting with STEMI and 15.2% with NSTEMI in a large representative sample of the German population during a longer phase of observation (January through May 2020). The maximum decline of NSTEMI presentations was detected in April 2020 (− 21.5% compared to 2019), followed by a slow recovery in May 2020, essentially mirroring SARS-CoV-2 infection rates in Germany. These observations were of particular interest as—opposed to other countries—Germany was not affected as strongly by the COVID-19 pandemic and a significant limitations of health-care resources did not become evident. This may explain why we found a milder decline compared to regions heavily affected by the disease outbreak [25] and was in line with reports from other less-affected areas reporting reductions of approximately 25% in acute cases. Interestingly, admissions for several cardiovascular and cerebrovascular emergencies started to increase again in May, suggesting a potential recovery after a significant drop in new daily COVID-19 cases and a liberalization of public restrictions in Germany.

Similar trends were observed for patients with acute cerebrovascular and peripheral vascular diseases. Using surrogate markers for the quantity of care, a large recent analysis reported a decrease of 39% in March 2020 in patients presenting with acute ischemic stroke to US hospitals [8]. The results of the current study confirmed declines in strokes and TIA during the pandemic in Germany, albeit less severe. Consistent with these observations, we detected a marked decline in the admission rates for acute limb ischemia during the pandemic. Whether this translates into increased disease severity and worse outcomes as others have suggested [26] will need to be investigated.

Additional information on age, gender, and comorbidities revealed patient populations to be similar before and during the pandemic, arguing against the notion that milder affected patients might particularly refrain from seeking medical care, thus shifting the severity of diseases. Likewise, treatment allocation of the patients admitted remained unchanged. Furthermore, in the current study, patients presenting to the emergency departments with acute cardiovascular or cerebrovascular disease had a similar chance to receive interventional or open-surgical therapy before and during the pandemic in Germany. This seems to be an indicator for an ongoing high-level medical therapy during this time period. Since rapid diagnosis and treatment of acute coronary syndromes are important to avoid further complications [27, 28], recently published results from Italy are alarming reporting a delay in coronary revascularization and increasing rates of case fatality and major complications in patients suffering from acute myocardial infarction during COVID-19 [14]. To guide the management of acute coronary syndromes and cardiovascular disease during the COVID-19 pandemic and to contain collateral damage, the European Association of Percutaneous Cardiovascular Interventions and German Cardiac Society recently issued comprehensive expert documents [29, 30].

The root causes for the observed decline in acute admissions for cardiovascular and cerebrovascular diseases remain unclear and may be multifactorial: avoidance of medical care due to patient-based anxiety and fear of contagion during the pandemic may play an important role. However, an attitude toward increased deferrals of less urgent cases by health-care personnel and lifestyle changes among other confounders during the time of extensive social restrictions need to be evaluated as well. Patient information and public education will be of paramount importance to contain collateral damages caused by delayed medical treatment for acute cardiovascular and cerebrovascular diseases.

Most previous reports on this topic used either surveys [31], selective multi-center data, or surrogate parameters (e.g., use of imaging software) [8] to quantity patient care. Population-based data investigating emergency admissions in this setting validly remain scarce. Nevertheless, the following limitations merit consideration. First, health insurance claims data were not primarily collected for research purposes and retrospective observational studies are unsuitable to prove underlying causal relationships. However, data from health insurance claims, as used in this analysis, may help to identify potential implications for both health-care professionals and regulators at an early stage. Second, while longitudinal data were available between 2008 and today, pandemic measures in Germany began in February 2020 with no relevant follow-up available. Therefore, future analyses investigating patients’ outcomes are required. This is particularly important for mortality, albeit differentiating COVID-19-related mortality from mortality due to other causes in the light of COVID infection will remain complex from insurance claims data. Third, admission diagnoses were used for current analyses in a small share of cases in May 2020 (0.6%) if the main diagnosis was not yet submitted. However, comorbidities were only analyzed for individuals with complete datasets available to avoid classification bias. Last, the BARMER sample included a relevant proportion of the entire population in Germany. A selection bias appeared unlikely and the BARMER cohort was comparable to other European countries in terms of comorbidities, age, and gender. In contrast to clinical registries or administrative data, the BARMER cohort was less affected by selection bias and includes all age groups, all hospitals, and all medical specialties. However, spatial analyses aiming for regional differences were not feasible in the existing dataset.

## Conclusions

In this large-scale retrospective analysis of health insurance claims, we observed a marked decrease of in-hospital admission rates for acute cardiovascular and cerebrovascular emergencies including myocardial infarction, acute limb ischemia, stroke, and transient ischemic attack during the COVID-19 pandemic in Germany. No changes were seen in these patients’ comorbidities and treatment allocations. The underlying root cause for these developments and subsequent implications on patient outcomes warrant further investigation. Public education will be important to contain collateral damage caused by delayed treatment for acute cardiovascular and cerebrovascular diseases.

## Electronic supplementary material

Below is the link to the electronic supplementary material.

Supplementary file1 (DOCX 16 kb)
