# Supplementary material for: Temporal trends in the presentation of cardiovascular and cerebrovascular emergencies during the COVID-19 pandemic in Germany: an analysis of health insurance claims
Source: Clin Res Cardiol. 2020 Aug 4;109(12):1540–8. doi: 10.1007/s00392-020-01723-9 (PMC7402080; doi:10.1007/s00392-020-01723-9)
Supplement: Supplementary file 1 — Supplementary file1 (DOCX 16 kb) [file 392_2020_1723_MOESM1_ESM.docx]

**Electronic Supplement**

Table 1: Coding criteria used for this retrospective observational study of health insurance claims. ICD: International Classification of Diseases. OPS: Operations and Procedure Coding.

| **1A) ST-segment elevation myocardial infarction (STEMI)** | |
| --- | --- |
| **Primary diagnosis / emergent presentation** | |
| **ICD 10 code** |  |
| I21.0 | Acute transmural myocardial infarction of anterior wall |
| I21.1 | Acute transmural myocardial infarction of posterior wall |
| I21.2 | Acute transmural myocardial infarction of other sides |
| I21.3 | Acute transmural myocardial infarction of unspecified side |
| **Invasive treatment** | |
| **OPS code** |  |
| 8-837 ff | Percutaneous coronary intervention |
| 5-361 ff | Coronary artery bypass graft surgery |
| 5-362 ff | Minimally invasive coronary artery bypass graft surgery |
| **1B) non-ST-segment elevation myocardial infarction (NSTEMI)** | |
| **Primary diagnosis / emergent presentation** | |
| **ICD 10 code** |  |
| I21.4 | Acute subendocardial myocardial infarction |
| **Invasive treatment** | |
| **OPS code** |  |
| 8-837 ff | Percutaneous coronary intervention |
| 5-361 ff | Coronary artery bypass graft surgery |
| 5-362 ff | Minimally invasive coronary artery bypass graft surgery |
| **2) Acute limb ischemia** | |
| **Primary diagnosis / emergent presentation** | |
| **ICD 10 code** |  |
| I74.0 | Embolism or thrombosis of the abdominal aorta |
| I74.1 | Embolism or thrombosis of the aorta, unspecified |
| I74.2 | Embolism or thrombosis of the upper extremities |
| I74.3 | Embolism or thrombosis of the lower extremities |
| I74.4 | Embolism or thrombosis of the extremities, unspecified |
| I74.5 | Embolism or thrombosis of the iliac arteries |
| I74.8 | Embolism or thrombosis, unspecified |
| I74.9 | Embolism or thrombosis of the arteries, unspecified |
| I70.23 | Peripheral arterial occlusive disease with ischemic rest pain |
| **Invasive treatment** | |
| **OPS code** |  |
| 5-380 | Embolectomy and thrombectomy |
| 5-381 | Endarterectomy |
| 5-382, 5-383, 5-384 | Transposition, Interposition of vessels |
| 5-388 | Suture of vessels |
| 5-393 | Arterial shunt |
| 5-394 | Revision of a vascular procedure |
| 5-395 | Patchplasty |
| 5-396 | Transposition of vessels |
| 8-836 | Percutaneous transluminal angioplasty |
| 8-83c | Other percutaneous intervention of arteries |
| 8-84 | Percutaneous angioplasty with stent |
| 5-862, 5-863, 5-864, 5-865 | Amputation |
| **2) Aortic rupture** | |
| **Primary diagnosis / emergent presentation** | |
| **ICD 10 code** |  |
| I71.04 | Aortic dissection with rupture, unspecified |
| I71.05 | Aortic dissection with rupture, thoracic |
| I71.06 | Aortic dissection with rupture, abdominal |
| I71.07 | Aortic dissection with rupture, thoracoabdominal |
| I71.1 | Aortic aneurysm with rupture, thoracic |
| I71.3 | Aortic aneurysm with rupture, abdominal |
| I71.5 | Aortic aneurysm with rupture, thoracoabdominal |
| I71.8 | Aortic rupture, unspecified |
| **Invasive treatment** | |
| **OPS code** |  |
| 5-384 | Open aortic surgery |
| 5-38a | Endovascular aortic surgery |
| **4A) Acute stroke** | |
| **Primary diagnosis / emergent presentation** | |
| **ICD 10 code** |  |
| I61 | Intracerebral hemorrhage |
| I63, I64 | Stroke |
| **Invasive treatment** | |
| **OPS code** |  |
| 8-020.8 | Thrombolysis |
| 8-836.80 | Mechanical thrombectomy |
| 5-380.0, 5-381.0, 5-382.0, 5-383.0, 5-393.0, 5-393.3, 5-395.0 | Carotid artery surgery |
| 8-84 | Percutaneous angioplasty with stent |
| **4B) Transient ischemic attack** | |
| **Primary diagnosis / emergent presentation** | |
| **ICD 10 code** |  |
| G45 | Transient ischemic attack |
| **Invasive treatment** | |
| **OPS code** |  |
| 8-020.8 | Thrombolysis |
| 8-836.80 | Mechanical thrombectomy |
| 5-380.0, 5-381.0, 5-382.0, 5-383.0, 5-393.0, 5-393.3, 5-395.0 | Carotid artery surgery |
| 8-84 | Percutaneous angioplasty with stent |
